# Automated age- and sex-specific volumetric estimation of regional brain atrophy: workflow and feasibility

**DOI:** 10.1007/s00330-020-07196-8

**Published:** 2020-08-27

**Authors:** Julian Caspers, Adrian Heeger, Bernd Turowski, Christian Rubbert

**Affiliations:** Department of Diagnostic and Interventional Radiology, University Düsseldorf, Medical Faculty, Moorenstr. 5, D-40225 Düsseldorf, Germany

**Keywords:** Atrophy, Brain, Gray matter, Magnetic resonance imaging, Neurodegenerative diseases

## Abstract

**Objectives:**

An automated workflow for age- and sex-specific estimation of regional brain volume changes from structural MRI relative to a standard population is presented and evaluated for feasibility.

**Methods:**

T1w MRI scans are preprocessed in a standardized way comprising gray matter (GM) segmentation, normalization, modulation, and spatial smoothing. Resulting GM images are then compared to precomputed age- and sex-specific GM templates derived from the population-based Nathan Kline Institute Rockland Sample, and voxel-wise z-maps are compiled. z-maps are color-coded and fused with the subject’s T1w images. The rate of technical success of the proposed workflow was evaluated in 1330 subjects of the Alzheimer’s Disease Neuroimaging Initiative (ADNI). Furthermore, medial temporal atrophy (MTA) was assessed using the color-coded maps and with the MTA visual rating scale in these subjects. Sensitivities and specificity of color-coded maps and MTA scale were compared using McNemar’s test.

**Results:**

One test dataset was excluded due to severe motion artifacts. Out of the remaining 1329 datasets, atrophy map generation was successful in 1323 ADNI subjects (99.5%). Sensitivity for AD diagnosis (71.4 % vs. 53.3%, *p* < 0.0001 for left; 70.4% vs. 55.3%, *p* < 0.0001 for right hemisphere) and for MCI (45.4% vs. 17.4, *p* < 0.0001 for left; 43.5% vs. 14.6%, *p* < 0.0001 for right hemisphere) based on medial temporal atrophy assessment in color-coded maps was significantly higher than for MTA visual rating scale, while specificity was lower (78.4% vs. 93.8%, *p* < 0.0001 for left; 79.4% vs. 95.8%, *p* < 0.0001 for right hemisphere). The workflow is named *veganbagel* and is published as open-source software with an integrated PACS interface.

**Conclusions:**

Automated brain volume change estimation with the proposed workflow is feasible and technically dependable. It provides high potential for radiologic assessment of brain volume changes and neurodegenerative diseases.

**Key Points:**

*• A workflow combining techniques from voxel-based morphometry and population-based neuroimaging data is feasible and technically highly dependable.*

*• The workflow is provided as open-source software, named veganbagel.*

*• Sensitivity of medial temporal atrophy assessment in atrophy maps from veganbagel exceeds the sensitivity of MTA visual rating scale for the diagnosis of Alzheimer’s disease.*

## Introduction

Normal aging is accompanied with decrease of brain volume [1]. The evaluation of deviations of regional brain volume beyond this physiological atrophy from cranial MRI is challenging and entails immense variations across readers [2]. Nevertheless, the detection of regional brain atrophy and accelerated brain aging is substantial for radiologic assessment, in particular in the diagnostic workup of neurodegenerative diseases.

Most neurodegenerative diseases are accompanied by specific atrophy patterns of the brain. For example, in Alzheimer’s disease (AD), the most prevalent neurodegenerative disease, a mesiotemporal as well as parietal atrophy pattern, is typically evident [3, 4]. Specific visual rating scales, such as the medial temporal atrophy (MTA) visual rating scale [5] or the posterior atrophy score of parietal atrophy [6], have increasingly gained importance to assess these atrophy patterns in a structured way in a clinical setting [7]. However, the assessment of these scales is to some degree still subjective and does not take the subject’s age and sex into account. On the other hand, using volumetric analysis tools to quantify brain atrophy in MRI data is often time-consuming and may require specialist software and expertise, which hinders their use in clinical practice [7].

Hence, an observer-independent procedure for reliable detection of brain volume changes with respect to the subject’s normative cohort that easily implements into radiologic workflows is desirable. We present an automated workflow for volumetric generation of age- and sex-specific atrophy maps relative to a standard population.

## Materials and methods

This study was approved by the local institutional ethics board. Subject data used in the preparation for this article was obtained from publicly available study samples, i.e., the enhanced Nathan Kline Institute (NKI) Rockland Sample [8] and the Alzheimer’s Disease Neuroimaging Initiative (ADNI) (http://adni.loni.usc.edu/). Written informed consent was obtained as part of these studies.

**Fig. 1:**
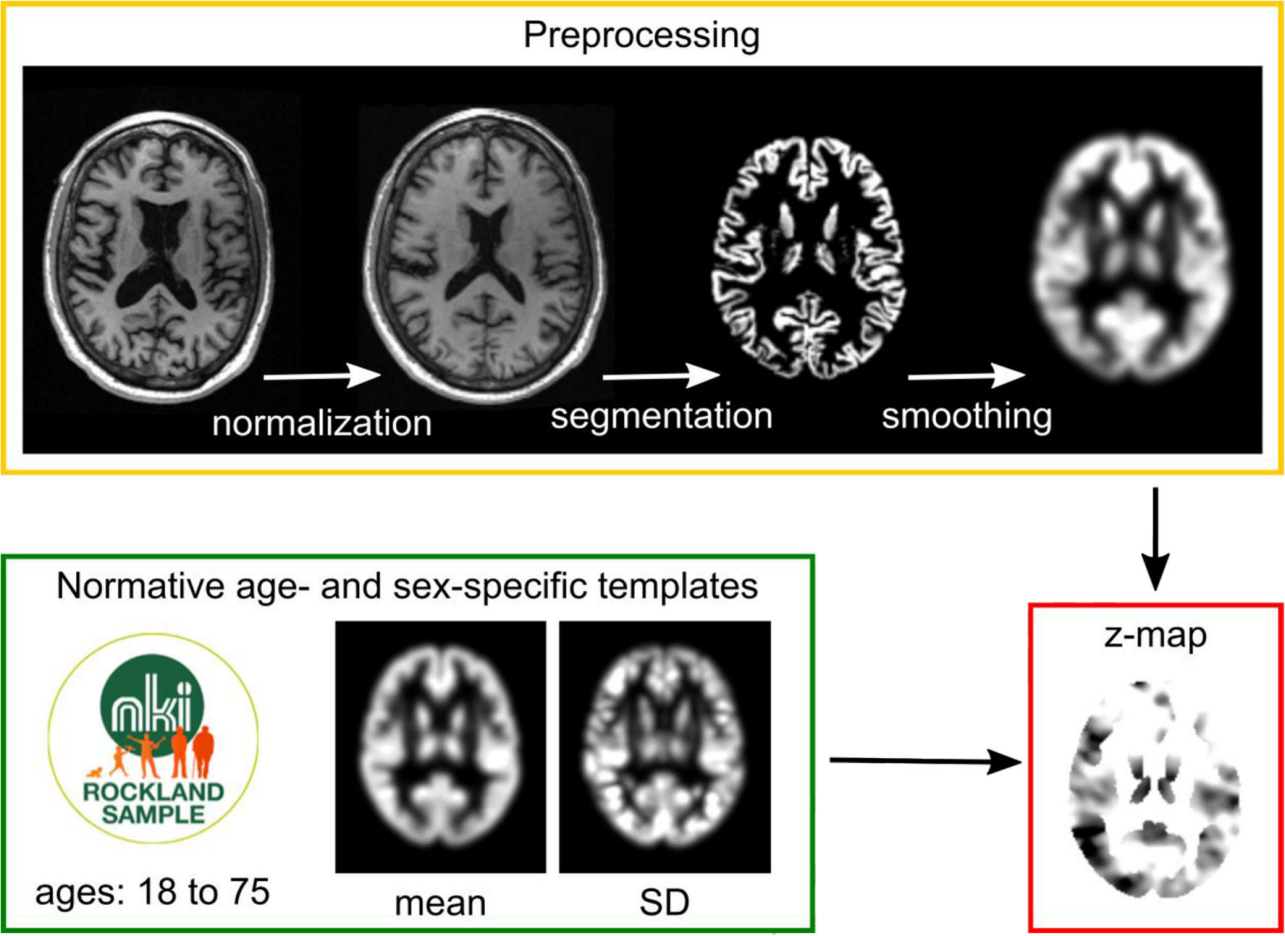
The veganbagel workflow. Standardized preprocessing (yellow box) of a 3D T1w MRI results in a normalized, smoothed gray matter map, which is compared to its age- and sex-specific gray matter templates (green box) to generate a z-map (red box). The z-map is afterwards color-coded and fused with the original T1w images

A workflow is proposed that estimates voxel-wise gray matter (GM) deviations from a normative age cohort in single subjects (Fig. 1). It is based on preprocessing methods established for voxel-based morphometry (VBM) [9, 10] and uses age- and sex-specific GM templates derived from a large population-based cohort to estimate GM volume deviations.

### Generation of normative gray matter templates

3D T1w cerebral MRIs (3T Siemens MAGNETOM Trio Tim, TR = 1900 ms, TE = 2.52 ms, TI = 900 ms, flip angle = 9°, FoV = 250 mm, matrix = 256x256, 176 slices, slice thickness = 1.0 mm) of 693 healthy subjects aged between 16 and 77 years from the publicly available, population-based enhanced NKI Rockland Sample were preprocessed in a standardized way: T1w images were segmented and normalized to MNI152 standard reference space [11] as implemented in the Computational Anatomy Toolbox (CAT12; http://www.neuro.uni-jena.de/cat/index.html) for SPM12 (https://www.fil.ion.ucl.ac.uk/spm/software/spm12/) in MATLAB (The MathWorks, Inc). The resulting GM images were scaled (modulated) by the amount of linear and non-linear transformations to preserve GM volumes rather than densities in each voxel [12]. The normalized and modulated GM maps were spatially smoothed with an 8-mm Gaussian kernel. Then, for each sex and age between 18 and 75, a mean and a standard deviation (SD) map was generated by calculating the voxel-wise mean and SD across the smoothed GM maps of all subjects aged between 2 years under to 2 years over the respective age.

### Estimation of volume changes of individual subjects

To estimate volume changes of a specific individual, his or her 3D T1w scan is preprocessed in the same way as for template generation, i.e., GM segmentation, normalization, modulation and smoothing. Then, voxel-wise *z*-values are generated from the resulting subject’s normalized GM map and the respective mean and SD template for his or her age and sex. A binary GM mask is applied to the z-map to restrict visualized changes to GM. The resulting z-map is transformed back into subject space, color-coded and fused with the structural MRI. To restrict atrophy map findings to relevant GM deviations from the normative cohort, only *z*-values above 2.5 or below − 2.5 are indicated in the color-coded maps.

### Evaluation

The implementation of the workflow was evaluated on the publicly available data of the ADNI database (http://adni.loni.usc.edu/). The ADNI was launched in 2003 as a public-private partnership, led by Principal Investigator Michael W. Weiner, MD. The primary goal of ADNI has been to test whether serial MRI, PET, other biological markers, and clinical and neuropsychological assessment can be combined to measure the progression of mild cognitive impairment (MCI) and early AD.

For evaluation purposes, 3D T1w cerebral MRIs of ADNI subjects under the age of 76 (204 AD patients, 645 MCI patients, 481 healthy controls) were processed with the proposed workflow to generate atrophy maps. ADNI scans were acquired in various 1.5-T or 3-T MRI scanners of different vendors (detailed specifications of scanning parameters can be found at [13] and http://adni.loni.usc.edu/methods/documents/mri-protocols/). The rate of technically successful atrophy map generation was assessed. Technical success was defined as follows: (1) the processing pipeline finished without error, (2) a color-coded atrophy map was created, and (3) the map was correctly aligned to the structural image; i.e., colored areas of relevant brain volume change were located in position of brain tissue and centered at GM. Furthermore, for all subjects with successful map generation, it was evaluated if a medial temporal atrophy pattern was observable; i.e., when any blue color was visible at the medial temporal region in the color-coded maps, indicating that a deviation of volume of at least 2.5 standard deviations below the mean volume is present. Additionally, the MTA visual rating scale [5] was obtained in these subjects. According to common practice, an MTA score ≥ 2 was considered pathological [5, 14]. The rate of positive/pathological findings in temporal atrophy assessment, i.e., the sensitivity for AD or MCI, as well as specificity was compared between the color-coded maps and MTA visual rating scale using McNemar’s test. Assessment of technical success, atrophy in color-coded maps, and MTA scores was performed by one reader (A.H.) with 3 years of experience in neuroimaging. Atrophy assessment in color-coded maps and with MTA scores was performed independently for each subject, i.e., at different time points and blinded to the result of the other modality. Statistics were conducted using R v3.6.3 (https://cran.r-project.org/).

## Results

The proposed workflow was implemented into a comprehensive software package, which allows for the fully automatic generation of atrophy maps from 3D T1w cerebral MRI scans and also provides a DICOM interface for direct communication with the PACS. In essence, a local server accepts DICOM images sent from the PACS or an MRI scanner, processes these images automatically, and exports the resulting atrophy map back to the PACS. Receiving and sending of DICOM files, as well as DICOM header operations, are handled using DCMTK (https://dicom.offis.de/, OFFIS e.V., Germany). Internal conversion from DICOM into Neuroimaging Informatics Initiative (NIfTI) file format is done using the established dcm2niix software (https://github.com/rordenlab/dcm2niix). The method, as well as the implemented software, will be referred to as *veganbagel* (*v*olumetric *e*stimation of *g*ross *a*trophy a*n*d *b*rain *age l*ongitudinally). The *veganbagel* software is implemented in an accessible scripting approach based on the Bourne-again shell (BASH), executable on most UNIX based systems, and is published as open source (https://github.com/BrainImAccs/veganbagel).

3D T1w datasets of 1330 subjects of the ADNI cohort were processed using the *veganbagel* software. Mean age of the sample was 68.9 (range 55–75). Six hundred eighty-one subjects (51.2%) were female. One AD dataset was excluded due to severe motion artifacts. Of the remaining 1329 scans, processing was technically successful resulting in the generation of an atrophy map in 1323 subjects (99.5%). Processing failed in 4 AD subjects (3 males, 1 female) and 2 MCI subjects (both female).

**Fig. 2:**
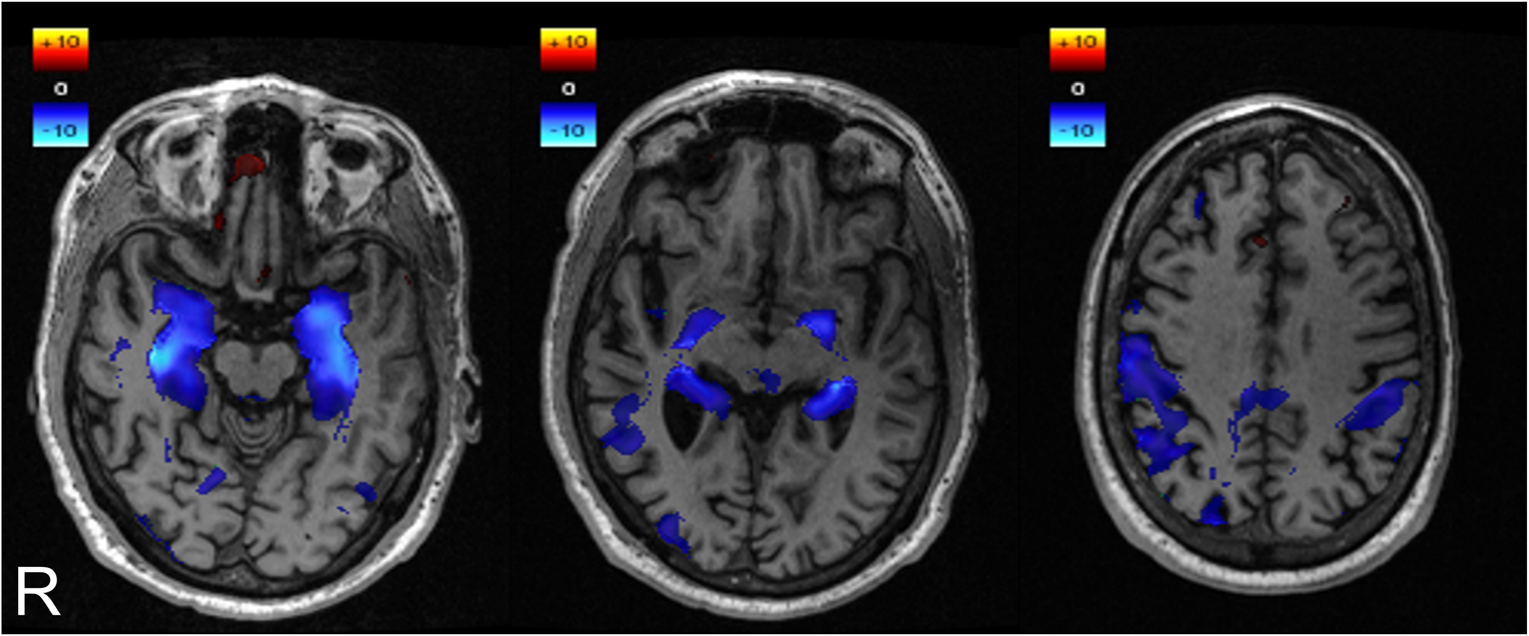
Atrophy map resulting from veganbagel of a 68-year-old male Alzheimer’s disease patient from the Alzheimer’s Disease Neuroimaging Initiative (ADNI). Three representative axial slices are shown. Color bars in the upper left corners indicate *z*-values of brain volume deviation from the respective sex and age norm

In the scans of AD patients with successful atrophy map generation, a temporoparietal atrophy pattern was observable in most patients (Fig. 2). Distinct medial temporal atrophy could be detected in the atrophy maps of 142 out of the 199 AD cases (71.4 %) in the left and 140 cases (70.4%) in the right hemisphere (Table 1). In comparison, MTA scores were pathological (≥ 2) in 106 cases (53.3%) on the left and 110 cases (55.3%) on the right side in those patients. These rates, i.e., sensitivity for AD, were significantly higher for atrophy maps than for MTA scores in both hemispheres (left: *p* < 0.0001; right: *p* < 0.0001). In MCI patients, a medial temporal atrophy pattern could be detected in the atrophy maps of 292 out of 643 MCI patients (45.4%) in the left and 280 (43.5%) in the right hemisphere, while MTA scores where pathological in 112 cases (17.4%) in the left and 94 cases (14.6%) in the right hemisphere (Table 2). These sensitivities for MCI significantly differed between both assessments (left: *p* < 0.0001; right: *p* < 0.0001). In healthy controls, atrophy maps indicated a medial temporal atrophy in 104 out of 481 cases (21.6%) in the left and 99 cases (20.6%) in the right hemisphere (Table 3). MTA scores where pathological in 30 cases (6.2%) in the left and 20 cases (4.2%) in the right hemisphere in healthy controls. The specificity for the atrophy maps (left 78.4%; right 79.4%) was significantly lower than that for MTA scores (left 93.8%, *p* < 0.0001; right 95.8%, *p* < 0.0001).

**Table 1:** Alzheimer’s disease. Fourfold tables specifying the rate of medial temporal atrophy findings in *veganbagel* and MTA visual rating scale for the left and right hemispheres in 199 Alzheimer’s disease patients from the Alzheimer’s Disease Neuroimaging Initiative (ADNI)

**Left hemisphere**		**MTA visual rating scale**	
		**MTA ≥ 2**	**MTA < 2**
*veganbagel*	Medial temporal atrophy	100	42
	No medial temporal atrophy	6	51
**Right hemisphere**		**MTA visual rating scale**	
		**MTA ≥ 2**	**MTA < 2**
*veganbagel*	Medial temporal atrophy	99	41
	No medial temporal atrophy	11	48

**Table 2:** Mild cognitive impairment. Fourfold tables specifying the rate of medial temporal atrophy findings in *veganbagel* and MTA visual rating scale for the left and right hemisphere in 643 mild cognitive impairment (MCI) patients from the Alzheimer’s Disease Neuroimaging Initiative (ADNI)

**Left hemisphere**		**MTA visual rating scale**	
		**MTA ≥ 2**	**MTA < 2**
*veganbagel*	Medial temporal atrophy	100	192
	No medial temporal atrophy	12	339
**Right hemisphere**		**MTA visual rating scale**	
		**MTA ≥ 2**	**MTA < 2**
*veganbagel*	Medial temporal atrophy	83	197
	No medial temporal atrophy	11	352

**Table 3:** Healthy controls. Fourfold tables specifying the rate of medial temporal atrophy findings in *veganbagel* and MTA visual rating scale for the left and right hemisphere in 481 healthy controls from the Alzheimer’s Disease Neuroimaging Initiative (ADNI)

**Left hemisphere**		**MTA visual rating scale**	
		**MTA ≥ 2**	**MTA < 2**
*veganbagel*	Medial temporal atrophy	26	78
	No medial temporal atrophy	4	373
**Right hemisphere**		**MTA visual rating scale**	
		**MTA ≥ 2**	**MTA < 2**
*veganbagel*	Medial temporal atrophy	18	81
	No medial temporal atrophy	2	380

## Discussion

We demonstrate a workflow for the generation of atrophy maps based on T1w cerebral MRI scans. This workflow, named *veganbagel*, allows for voxel-wise semiquantitative assessment of GM volume deviations of a single subject relative to his or her normative age cohort. The implementation of the workflow is published as open source and can assist radiologists in the assessment of brain atrophy and neurodegenerative diseases. An evaluation of feasibility of the implemented software shows a very high technical reliability and good results regarding the detection of disease-typical atrophy patterns in AD.

The VBM methodology our approach is based on has been proven to reliably indicate gray matter alterations associated with normal aging and neurodegenerative diseases [12, 15]. With the implemented fully automatic approach, these valuable techniques established in imaging neurosciences are now translated into the clinical field and easily accessible for radiologists.

The evaluation of *veganbagel* on ADNI data indicates that the method is feasible and technically very dependable (99.5%). Furthermore, we could show that atrophy maps made clinically meaningful patterns apparent, as most of the maps in AD patients clearly revealed a medial temporal atrophy pattern. Indeed, the rate of *veganbagel* indicating this AD typical pattern, i.e., its sensitivity for AD, clearly exceeded the rate of pathological scores of the established MTA visual rating scale in our sample (71.4% vs 53.3% on the left, 70.4% vs 55.3% on the right side). The rather low sensitivity of the MTA scale and—while higher—also of *veganbagel* is probably due to the fact that most AD patients in ADNI are at very early stages of disease [16]. In fact, the current sensitivity of MTA scores is only slightly lower than that in other reports investigating MTA in mild to moderate AD [17]. Sensitivity for MCI was likewise significantly higher for *veganbagel* compared to MTA (45.4% vs. 17.4, on the left, 43.5% vs. 14.6% on the right side). However, its specificity was substantially lower (78.4% vs. 93.8% on the left, 79.4% vs. 95.8% on the right side). Nevertheless, in a screening setting, a high sensitivity for diseases is of particular interest, so that the higher sensitivities of *veganbagel* for AD and MCI compared to the current state of the art visual rating scale make it potentially valuable as a screening tool.

There are several commercially available software products for brain volumetry on the market, e.g., NeuroQuant (CorTechs Labs), Neuroreader (Brainreader Aps), or icobrain (icometrix). However, these tools are largely based on surface-based segmentations measuring the total volume of predefined regions of interests. The output of these tools is usually an extensive datasheet of numbers and metrics specifying volume aberrations in each region of interest, which barely leaves space for image-based interpretations by the radiologist. Furthermore, besides the fact that they are not free to use, the way the algorithms work is a black box to the user. *veganbagel* offers a transparent open-source solution for semiquantitative volumetric analysis of brain MRI relative to the normative age cohort and builds up on established methods known from imaging neurosciences. Compared to the aforementioned number-centric reports of commercial software solutions, the voxel-wise volumetry and color-coded cross-sectional atrophy maps provided by *veganbagel* much more align with radiological workflows, and the privilege of interpretation and diagnosis based on images is still with the radiologist. The server-based implementation with a full DICOM interface provides the opportunity to automatically receive atrophy maps into the PACS that the radiologist is used to without the need for any user interaction or any additional software on the radiologist workstation. We deliberately decided to publish *veganbagel* as open source and allow access to this tool to the radiologic and scientific community for free. By this means, we hope that *veganbagel* grows in the community and can further improve to be a valuable assistant tool for radiologists.

A potential limitation of the approach might be the varying number of subjects underlying the normative templates for specific ages. We chose the enhanced NKI Rockland sample for the basis of these templates, as it provides coherent MRI data of sufficient quality from subjects quite equally distributed over all ages of the whole lifespan [8]. Currently, we limited the age range of generated templates to adults over the age of 18 and under the age of 76 because of increasingly sparser data for higher ages within the sample. It has to be acknowledged that volumetry from the normative sample might not be generalizable to other demographics or populations, as there is evidence for culture-related differences in regional brain volume [18]. The Rockland sample was shown to be representative regarding ethnicity and socioeconomic status at least for the US population [8]. Furthermore, the modular integration of age- and sex-specific GM templates in *veganbagel* allows to easily supplement or exchange the existing data basis of the normative age cohort, e.g., for adding data from other population-based studies or tailoring the normative data to local needs. The method has been developed for 3D T1w MRI scans. The use of imaging parameters substantially different from the underlying normative sample might potentially lead to inaccuracies. However, with the evaluation of the ADNI sample, which incorporates various scanner types and imaging sequences, we provide evidence that *veganbagel* performs very robustly with regard to different scanning parameters, scanners, and vendors. Obviously, volumetric accuracy will be coarser when lower image resolution is used. 2D imaging is not expected to be suitable for volumetry.

By providing a semiquantitative orientation of GM deviations relative to the age norm and given the convincing results of the first evaluation of the tool, *veganbagel* can substantially add value to the radiologist’s assessment of regional brain volume changes and neurodegenerative disorders. However, more extensive evaluations of the tool are needed to prove these claims and to provide conclusive insights on its impact, which is in the scope of our ongoing research.

## Abbreviations

AD: Alzheimer’s disease
ADNI: Alzheimer’s Disease Neuroimaging Initiative
GM: Gray matter
MCI: Mild cognitive impairment
MTA: Medial temporal atrophy (visual rating scale)
NKI: Nathan Kline Institute
SD: Standard deviation
VBM: Voxel-based morphometry
